# *GPNMB* methylation: a new marker of potentially carcinogenic colon lesions

**DOI:** 10.1186/s12885-018-4903-7

**Published:** 2018-11-06

**Authors:** Hassan Ashktorab, Hamed Rahi, Mehdi Nouraie, Babak Shokrani, Edward Lee, Tahmineh Haydari, Adeyinka O. Laiyemo, Peter Siegel, Hassan Brim

**Affiliations:** 1 Department of Medicine, Department of Pathology and Cancer Center, Howard University College of Medicine, 2041 Georgia Avenue, N.W, Washington, D.C, 20060 USA; 2 Division of Pulmonary, Allergy and Critical Care Medicine, Department of Medicine, University of Pittsburgh, Pittsburgh, USA; 3 Goodman Cancer Research Centre, Department of Medicine, McGill University, Montréal, Québec, Canada

**Keywords:** GPNMB, Colon adenoma, African Americans

## Abstract

**Background:**

Epigenetic plays an important role in colorectal neoplasia process. There is a need to determine sound biomarkers of colorectal cancer (CRC) progression with clinical and therapeutic implications. Therefore, we aimed to examine the role and methylation status of Glyco Protein Non-Metastatic GPNM B (GPNMB) gene in normal, adenoma and CRC in African American (AA) patients.

**Methods:**

The methylation status of 13 CpG sites (chr7: 23287345–23,287,426) in *GPNMB* gene’s promoter, was analyzed by pyrosequencing in human CRC cell lines (HCT116, SW480, and HT29) and microdissected African American paraffin embedded samples (20 normal, 21 non-advanced adenoma (NA), 48 advanced adenoma (AD), and 20 cancer tissues. GPNMB expression was analyzed by immunohistochemistry (IHC) on tissue microarrays (TMA). Correlations between GPNMB methylation and expression with clinicopathological features were analyzed. GPNMB functional analysis was performed in triplicates using cell proliferation, migration and invasion assays in HCT116 colon cell line after stable transfection with a GPNMB-cDNA expression vector.

**Results:**

GPNMB methylation was lower in normal mucosa compared to CRC samples (1/20 [5%] vs. 18/20 [90%]; *P* < 0.001). AD also had a significantly higher GPNMB methylation frequency than normal colon samples (42/48 [88%] vs 1/20 [5%]; *P* < 0.001). GPNMB was more frequently methylated in AD than in matched normal mucosa from three patients (3/3 [100%] vs 1/3 [33.3%]; *P* < 0.001). The frequency of GPNMB methylation in NA differed significantly from that in the normal mucosa (16/21 [76%] vs 1/20 [5%]; *P* = 0.008). There was statistically significant correlation of higher methylation at advanced stages and lower methylation at stage 1 CRCs (*P* < 0.05). In agreement with these findings, GPNMB protein expression decreased in CRC tissues compared with AD and NA colon mucosa (*p* < 0.05). GPNMB overexpression in HCT116 colon cancer cell line decreased cell proliferation [(24 h, *P* = 0.02), (48 h, *P* < 0.001, 72 h, *P* = 0.007)], invasion (*p* < 0.05) and migration (*p* > 0.05) compared to the mock-transfected cells.

**Conclusion:**

Our data indicate a high methylation profile leading to a lower GPNMB expression in adenoma and CRC samples. The functional analysis established GPNMB as a potential tumor suppressor gene. As such, GPNMB might be useful as a biomarker of adenomas with high carcinogenic potential.

## Background

Colorectal Cancer (CRC) is one of the most leading causes of cancer-related mortality. Nearly 130,000 US residents are diagnosed with CRC every year. As of 2014, approximately one third of these patients died from the disease [1]. The impact of CRC on African Americans (AAs) is higher than the general population, both in incidence and mortality [1]. During the period from 2000 to 2009, CRC incidence declined by 1.9% and 2.1% annually among AA men and women, respectively, vs. 3.5% and 3% in Caucasian men and women, respectively [2]. CRC aggressiveness in AAs, as well as low screening rates, have been proposed as probable reasons for the observed disparities. In 2010, 56% of AAs and 62% of Caucasians, were reported to have colorectal cancer screening test [3]. Unique features of the disease in AAs include: a greater prevalence of right-sided lesions [4, 5], that are genetically different from rectosigmoid lesions in terms of hyperploidy and allelic changes [6], different karyotype characteristics of malignant cells in rectal adenocarcinomas, greater consumption of saturated fat, smoking, and alcohol consumption [7].

Colon polyps are also prevalent in 30–40% of the adult population in the United States [8]. Differences in cancer prevalence and recurrence have been reported between high risk AAs and Caucasians [8, 9]. Screening results indicate that AAs are at higher risk of developing cancer at an earlier age (> 40 years) than what the literature suggests (> 50 years) [10]. AA patients have worse survival rates among various populations after a diagnosis with similar cancer [11], specifically in terms of metastatic progression and response to chemotherapy [12]. The underlying etiology of different patterns of CRC incidence, mortality, and survival rates between these racial and ethnic populations has not yet been explained, and appears to be complex in origin [13]. The presence of various molecular mechanisms and pathways of colon carcinogenesis highlights the heterogeneous characteristic of CRCs [14].

Number of candidate genes, involved in cancer genesis were defined as genetically altered in the process. This number is considerably lower than the 500 genes that were identified through a cancer gene census. As such, they suggested that the other genes are epigenetically altered [15]. Study recommended through a whole human transcriptome microarray analysis that more epigenetic than genetic events are involved in tumorigenesis in any specific tumor [16]. Schuebel et al. have previously established a list of 13 candidate cancer genes that are predominantly methylated in CRC tumors. We have investigated the methylation status of these genes in a comparative study of AA and Caucasian CRC patients that led to 3 markers that were primarily and predominantly methylated in AAs, namely CHD5, ICAM5 and GPNMB [17]. We have further analyzed CHD5 function in-vitro and established its tumor suppressor gene status [18].

GlycoProtein Non-metastatic Melanoma B (*GPNMB*) was discovered in 1995, through a study on two melanoma cell lines with low and high metastatic abilities. The authors determined and characterized GPNMB cDNA that was highly expressed in the low metastatic cell line [19]. *GPNMB* is located on the short arm of chromosome 7 (7p15) and known as neurokinin-1 type, hematopoietic growth factor inducible (HGFIN) [20]. It is a type I transmembrane protein that has three unique domains: an extracellular, a transmembrane, and a cytoplasmic one. The extracellular domain consists of two regions with different properties, an integrin-binding motif (RGD) and a polycystic kidney disease (PKD) domain. The cytoplasmic tail has two functionally different parts including a dileucine-based sorting signal and a half immunoreceptor tyrosine-based activation motif (hemITAM) [21].

Here, we present a detailed analysis of *GPNMB* methylation status, expression and function in pre-neoplastic and neoplastic clinical specimens and in colon cancer cell lines to shed light on the potential role of this gene’s methylation in colorectal tumorigenesis.

## Methods

### Human participants

*GPNMB* promoter methylation was examined in microdissected African American paraffin embedded tissue samples (*n* = 109) with 20 normals, 21 non-advanced adenomas, 48 advanced adenoma, and 20 cancers collected at Howard University Hospital, between 2011 and 2012. The study protocol was approved by the Institutional Review Board (IRB, 06-MED-39) and informed consent forms were obtained from all patients study participants.

### DNA isolation

Genomic DNA was extracted from fresh frozen parafim embeded (FFPE) blocks using MO BIO kit (Carlsbad, CA) extraction according to the manufacturer’s protocol. Genomic DNA from human colon cancer cell lines (HCT116, SW480, and HT29) was extracted using QIAGEN AllPrep (Germantown, MD) DNA/RNA/Protein kit.

### Sodium bisulfite modification

Sodium bisulfite conversion was carried out on 300 ng genomic DNA isolated from the FFPE block tissue samples and colon cancer cell lines using the ZYMO EZ-DNA Methylation-Gold Kit (Irvine, CA). First, 130 μl of the CT conversion reagent was added and mixed to 20 μl of DNA solution (300 ng). Then, the DNA samples underwent the conversion in a thermal cycler under the following steps; Denaturation at 98 °C for 10 min, cytosine deamination at 53 °C for 30 min, followed by 8 cycles of 53 °C for 6 min and 37 °C for 30 min. Purification was carried out by adding a mixture of 600 μl of M-Binding buffer and bisulfite converted-DNA samples into a Zymo-Spin IC column (Irvine, CA). After centrifugation at > 10,000 g for 30 s, 100 μl of M-Wash buffer was added directly to the column. Samples were centrifuged at full speed for 30 s. To neutralize and remove the bisulfite adduct from the uracil ring (uracil sulphonate), 200 μl of M-Desulphonation buffer was added to the column and incubated at room temperature for 20 min. The columns were centrifuged for 1 min. Washing the column with 200 μl of M-Wash Buffer was done twice by centrifugation at full speed for 30 s. To elute the modified DNA, the column was placed in a fresh 1.5 microcentrifuge tube and 15 μl of M-Elution buffer, equilibrated to 65 °C, was added directly onto the membrane. The column was incubated for 5 min at room temperature and centrifuged at > 10,000 g for 1 min. The modified DNA was stored at − 20 °C.

### Pyrosequencing

Sodium bisulfite modified DNA was amplified using the forward and biotinylated reverse *GPNMB* specific primers (EpigenDx: ADS492; Hopkinton, Massachusetts) to establish the methylation status of 13 CpG sites (chr7:23287345–23,287,426) in GPNMB promoter. A total of 45 cycles were used for all PCR reactions with annealing temperature of 57 °C. The PCR products were visualized by electrophoresis on 1.5% agarose gel. PCR products (10 μl) were used in duplicates for pyrosequencing. Preparation of pyrosequencing reactions was performed and data analyzed as recommended by manufacturer (QIAGEN, Germantown, MD).

### Immunohistochemistry analysis

A 1 mm tissue cores were collected from 30 normal, 72 pre-neoplastic lesions and 43 cancers and used to build Tissue Microarrays (TMAs) in this study. Positive and negative non-colon controls were also spotted on the TMAs. Slides were cut from the generated TMAs. De-paraffinization/hydration of the TMAs was performed as follows: two xylene (5 min each), followed by two 100% ethanol washes (5 min each), followed by 95% ethanol, 70% ethanol, 50% ethanol, 30% ethanol, followed by H_2_O and a TBST wash for 5 min on a shaker. Immunohistochemical staining was done according to standard procedures using a polyclonal goat anti-GPNMB antibody (1:500 dilution; R&D Systems) and a HRP-conjugated donkey anti-goat secondary antibody (1:500 dilution; Jackson ImmunoResearch Laboratories; West Grove, PA). Sections were developed with 3–3-diaminobenzidine-tetrahydrochloride (DAB) and counterstained with hematoxylin. The immunostained slides were evaluated by two expert gastrointestinal pathologists (E.L, B.S). Both intensity and percentage of staining were used to evaluate and compare samples.

### Cell cultures and transfections

Colorectal cancer cell line, HCT116, was used for this study. HCT116 was selected because *GPNMB* was highly methylated, and its expression was reduced. HCT116 cells were maintained in McCoy’s medium with L-Glutamin and NaHCO_3_ supplemented with 10% fetal bovine serum (FBS) and Penicillin/Streptomycin (Life technologies; Carlsbad, CA). The pEF1-GPNMB vector and empty vector were used for transfection (Goodman Cancer Research Center, Montreal, Quebec). About 1 × 10^4^ cells/well in 24 well plates were seeded 24 h. before transfection in MEM medium without antibiotics. The cells were transfected with 0.6 μg of plasmid DNA and 1.2 μl of Lipofectamine 2000 (Invitrogen; Carlsbad, CA) per well diluted in 50 μl of OPTI-MEM medium (Invitrogen) without serum and antibiotics. After 24 h. transfection, the medium was changed. The cells were then treated with G418 at gradual concentrations ranging from 250 to 1000 ng/ml medium after 48 h. of transfection to select for transfects. The colonies were selected at the highest G418 concentration that was 750 ng/ml, isolated after 2 weeks and regrown in fresh medium for further characterization.

### Proliferation assay

For cell pproliferation, transfected HCT116 were used with CellTiter 96 Aqueous kit (Promega; Madison, WI) following the manufacturer’s instructions. Cells were seeded onto 96 well plates (5000 cells per well), and cell numbers were counted 24, 48, and 72 h later (six wells per time point). The absorbance at 490 nm was recorded using a 96 well plate reader.

### Migration and invasion assays

Migration and invasion assays were performed using CytoSelect 24-well. The cell migration and invasion assays (8 μm, colorimetric format) from Cell Biolabs, Inc. (San Diego, CA), were performed following the manufacturer’s instructions. Briefly, 2 × 10^5^ HCT116 cells in McCoy’s containing 1% FBS were seeded into the upper chamber of each well, and McCoy’s medium containing 20% FBS was placed in the lower chamber. After 48 h of incubation, the transwells were disassembled and the membranes that separated the upper and lower chamber of each transwell were fixed with methanol and stained with 1% toluidine blue in 1% borax. The cells on the lower surface of the membrane were counted under a light microscope.

### Statistical analyses

Kruskal-Wallis test was chosen for analyzing continuous data. The effect of gender, location of tumor, and disease stage on methylation status was investigated by Pearson chi-square (X^2^) analysis. To study the correlation between GPNMB expression and methylation level, Spearman’s rank correlation coefficient, a nonparametric measure of statistical dependence was used. Student’s t-test was used to analyze cell proliferation, migration, and invasion assays data. All *p* values are two-sided. *P* value less than 0.05 were considered statistically significant.

## Results

### Clinico-pathological characteristics of patients

The mean age (SD) for cancer patients was 66.4 (20.4) years while it was 63.3 (15.0) years for the healthy patients in this study (Table 1). There was no significant difference in gender or age between the two analyzed groups (Table 1). A total of 62% tumors were located in the right colon, respectively. Rectal cases accounted for 8%. Most tumors were at advanced stages with 26% at stage II, and 42% at stage III + IV. The 21 non-advanced adenoma (7 females/14 males) and 48 advanced adenoma (24 females/24 males) had a mean age (SD) of 62.8 (8.4) years and 60.2 (10.9), respectively (Table 1).

**Table 1:** Clinical and demographical features of collected samples including normal, adenoma, advanced adenoma, and colon cancer in African American cases for validation of the GPNMB methylation status

Type	Normal (*n* = 20)	Adenoma (*n* = 21)	Adv. Adenoma (*n* = 48)	Cancer (*n* = 20)	*P* value
Mean age (SD)	63.3 (15.0)	62.8 (8.4)	60.2 (10.9)	66.4 (20.4)	*P* = 0.09
Gender (%)					
Male	10 (50)	14 (66)	24 (50)	11 (55)	*P* = 0.6
Female	10 (50)	7 (33)	24 (50)	8 (45)	
Location (%)					
Proximal	7 (35)	16 (76)	30 (63)	12 (58)	*P* = 0.3
Distal	9 (45)	4 (19)	13 (27)	6 (32)	
Rectum	0	1 (5)	5 (10)	2 (10)	
Unknown location	4	0	0	0	

### Methylation status of *GPNMB* gene in CRC, normal tissues and cell lines

**Fig. 1:**
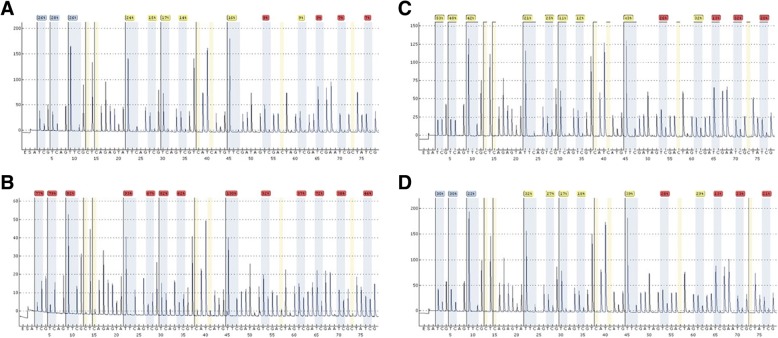
Pyrogram patterns for each type of samples (**a**. normal, **b**. cancer, **c**. advanced adenoma, and **d**. adenoma) methylation status of target 13 CpG sites (chr7: 23287345–23,287,426) in *GPNMB* gene. Below the pyrogram is the nucleotide dispensation order

The methylation status of 13 CpG promoter sites (chr7: 23287345–23,287,426) in *GPNMB* was measured by pyrosequencing using primers designed by EpigenDx primers were first tested on three human colorectal cancer cell lines (HCT116, HT29, and SW480) and one male normal blood DNA by pyrosequencing. *GPNMB* CpG sites at the promoter region were highly methylated in the three colon cancer cell lines while it was not methylated in the blood DNA. As a result, HCT116 DNA was used as positive control for *GPNMB* methylation (89%) in this study. Normal blood DNA was used as negative control with an average methylation level of 4.7%. GPNMB methylation was then tested in clinical samples of which the characteristics are given in Table 1. The frequency of *GPNMB* methylation was significantly lower in the normal mucosa samples than in the CRC tissues (1/20 [5%] vs 18/20 [90%]; *P* < 0.001), (Table 2 and Fig. 1).

**Table 2:** The frequency of *GPNMB* promoter methylation in normal, adenoma, and colon cancer tissues. Methylation frequency is presented as the number of methylated samples divided by the total number of samples analyzed (%)

Samples	Normal tissue	Adenoma	Adv. adenoma	Colon cancer
Methylation frequency	1/20 (5%)	16/21 (76%)	42/48 (88%)	18/20 (90%)
Mean Methylation levels	19.2	32.0	42.3	43.1
Adenoma	*P* < 0.001	–	–	–
Adv. adenoma	*P* < 0.001	*P* = 0.008	–	–
Colon cancer	*P* < 0.001	*P* = 0.020	*P* = 0.5	–

*P* values (two-sided) were generated by using chi-square test. *P* values are Bonferroni adjusted, and the cutoff for statistical significance is *P* ≤ 0.05

### Methylation status of *GPNMB* gene in pre-cancerous lesions

**Fig. 2:**
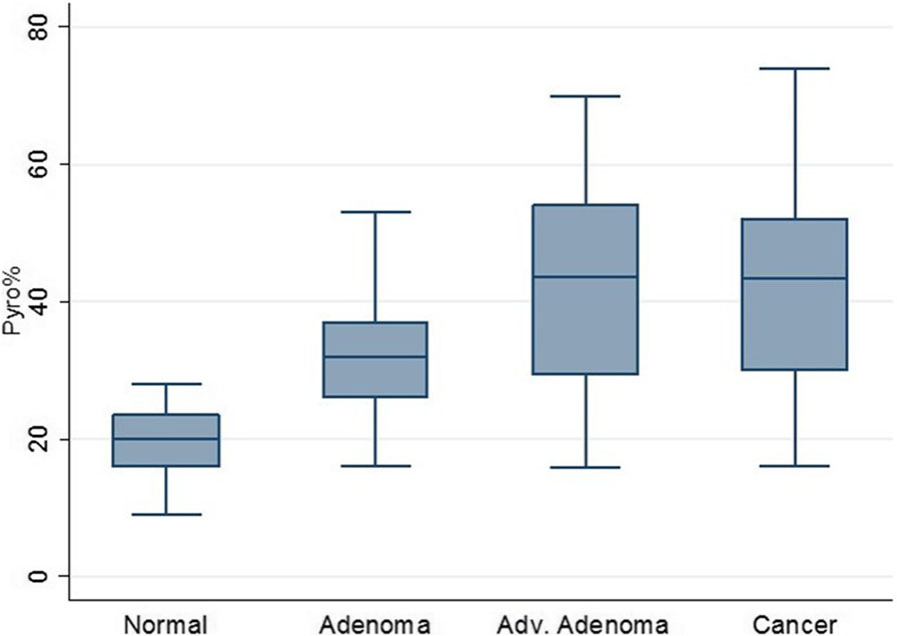
GPNMB methylation levels at different stages of colon carcinogenesis including normal, non-advanced, advanced adenoma, and colon cancer

Advanced adenomas (> 1 cm with villous component and/or high grade dysplasia) samples had a significantly higher *GPNMB* methylation frequency than normal colon samples (42/48 [88%] vs. 1/20 [5%]; *P* < 0.001) (Table 2 and Fig. 1c). Also, *GPNMB* was more frequently methylated in advanced adenoma than in matched normal mucosa from three patients (3/3 [100%] vs. 1/3 [33.3%]; *P* < 0.001). Finally, the frequency of *GPNMB* methylation in non-advanced adenoma differed statistically from that in the normal mucosa (16/21 [76%] vs. 1/20 [5%]; *P* < 0.001) (Table 2 and Fig. 1d). The comparison of methylation levels between non-advanced adenoma and advanced adenoma was statistically significant (16/21 [76%] vs. 42/48 [88%]; *P* = 0.012) (Table 2 and Fig. 2). The difference of *GPNMB* methylation in advanced adenoma and colorectal cancer tissues was not statistically significant (42/48 [88%] vs. 18/20 [90%]; *P* = 0.88) (Table 2 and Fig. 2). The difference between non-advanced adenoma and CRC tissues methylation frequencies was statistically non-significant (*P* = 0.2). *GPNMB* gene methylation was gender, location, and age independent.

### Cancer stage and *GPNMB* methylation

**Fig. 3:**
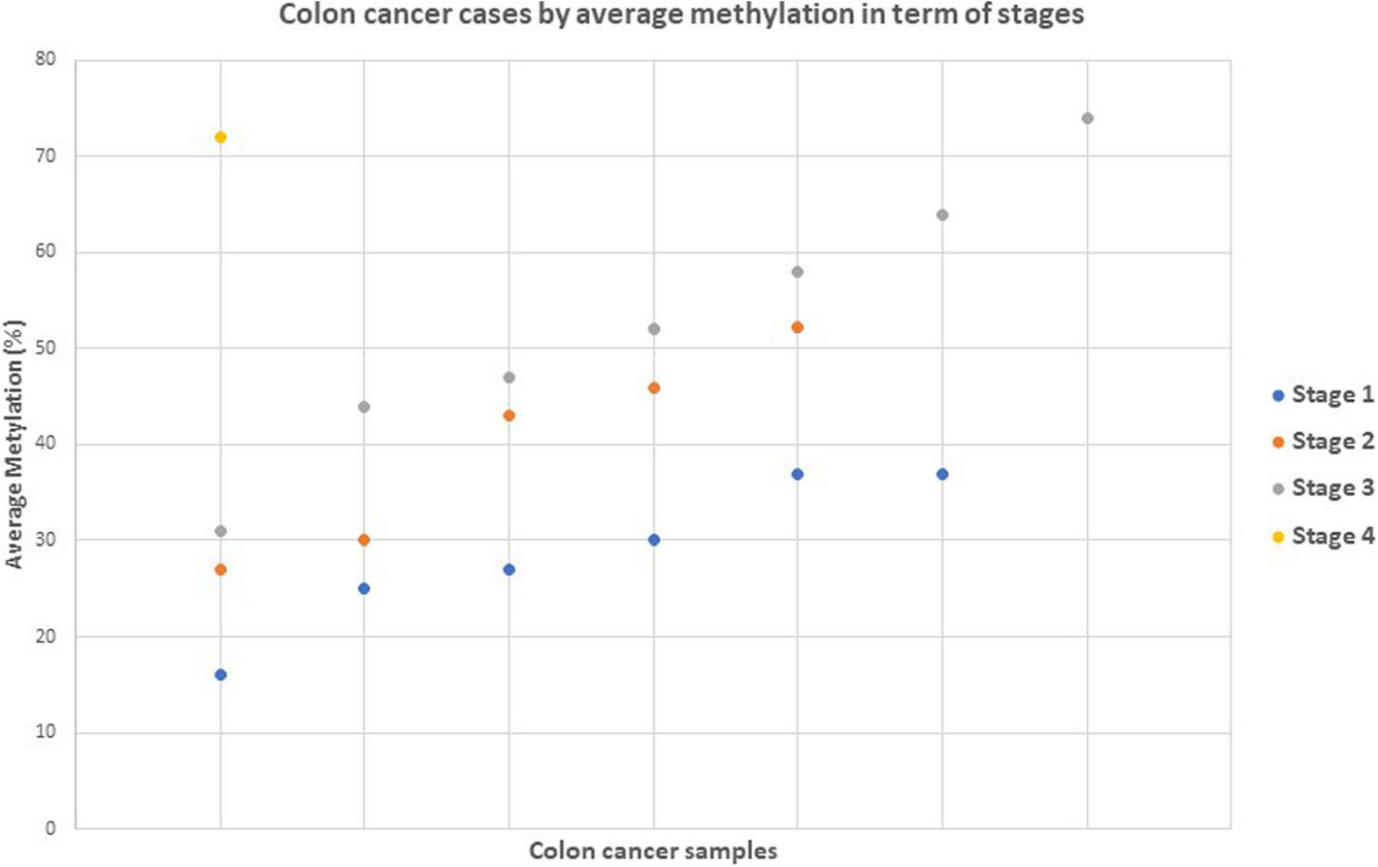
Methylation frequencies (%) of *GPNMB* in different stages of cancer. *GPNMB* methylation was statistically significant for advanced tumor stages (III + IV) (*P* < 0.012). *P* values (two-sided) were generated by using chi-square test. *P* values are Bonferroni adjusted, and the cutoff for statistical significance is *P* ≤ 0.05

*GPNMB* displayed lower methylation at stage 1 and higher methylation at more advanced cancer stages. There was a statistically significant increase in methylation for advanced cancer stages III-IV (*P* = 0.012). Also, there was an association between the increase of *GPNMB* methylation and the potential for progression to invasive stage adenocarcinomas. *GPNMB* was methylated at a rate of 73%, 86%, and 89% at stage I, II and (III + IV), respectively (Fig. 3).

### GPNMB expression profile

**Fig. 4:**
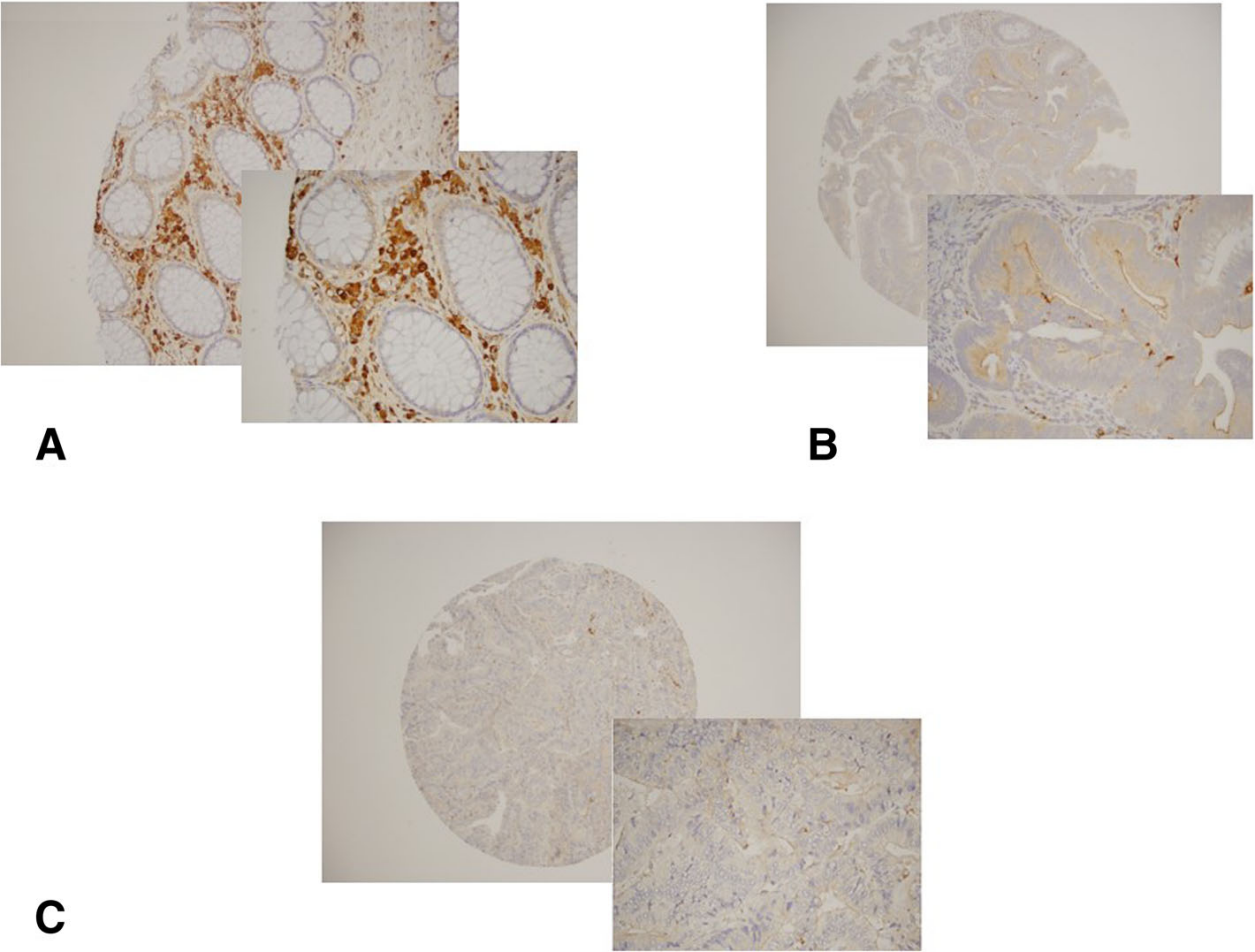
Immunohistochemical staining of GPNMB in human tissue microarray. 1 mm core were used from each patient’s FFPE Block on the TMAs. **a** Positive GPNMB staining of brown color (DAB) represented in membrane in normal colon biopsies, some staining was also noted in the stroma. **b** Moderate cytoplasmic membrane staining marked in biopsy specimens from advanced adenoma. **c** CRC patients showed absence of GPNMB staining in the malignant cells. Statistical analysis was performed using the chi-square test

To establish a correlation between DNA methylation and GPNMB expression, immunohistochemistry (IHC) in normal, pre-neoplastic lesions, and CRC tissue microarrays (TMA) was performed. The expression level of GPNMB in TMAs containing 43 CRCs, 72 pre-neoplastic lesions as well as 30 normal tissues were determined. Liver, lung, spleen, and kidney tissues were used as controls in the TMAs. The mean age (SD) for normal, pre-neoplastic lesions, and CRC tissues in TMAs was63 (14.6) years, 59 (8.9) years, and 66 (13.2) years, respectively (Table 3). There were no statistically significant differences for age and gender (Table 3). In normal colonic biopsy specimens, GPNMB expression was primarily in the cytoplasmic membrane, but also noticed in the stroma. Moderate to weak membrane staining was observed in pre-neoplastic and cancer samples on the TMAs (Fig. 4). After calculating the staining intensity in cells that positively stained for GPNMB, there was no significant difference between cancer, adenoma, and normal specimens.

**Table 3:** Clinical and demographical characteristics of collected samples from normal, pre-neoplastic lesions, and colon cancer African American patients included in the GPNMB IHC TMA study

Type	Normal (*n* = 30)	Pre-neoplastic lesions (*n* = 72)	Cancer (*n* = 43)	*P* value
Mean age (SD)	62.7 (14.6)	59.0 (8.9)	66.3 (13.2)	*P* = 0.06
Gender (%)				
Male	13(43)	38(53)	21(49)	
Female	17(57)	34(47)	22(51)	*P* = 0.7
Location (%)				
Proximal		36 (56)	14(35)	
Distal		28 (44)	26(65)	*P* = 0.035

### Cancer stages and *GPNMB* gene expression profile

Among 43 CRC cases available for analysis, the number of subjects with stage I, II, and (III + IV) were 14 (32.5%), 14 (32.5%) and 15 (34.8%), respectively. Expression levels at these different stages were calculated and interestingly displayed statistically significant differences with lower expression at advanced cancer stages (*P* < 0.05). This finding suggests lower expression of GPNMB might drive advancement of cancer stage. Similar findings were also observed with GPNMB methylation that might directly lead to loss of gene expression in late stage disease (Table 4).

**Table 4:** GPNMB IHC showed different expression profiles at different tumor stages, with statistically significant differences. Lower expression at advanced stages including II and (III + IV) was reported

Stage	I	II	III + IV	Total
Staining				
Moderate (%^a^)	8(57)	3(21)	5(33)	16(37)
Weak (%^a^)	6(43)	11(79)	10(67)	27(63)
Total (%)	14 (33)	14 (33)	15 (35)	43 (100)

^a^ From column

### Negative correlation between GPNMB expression and methylation profile in advanced adenoma and CRC

To investigate the association between *GPNMB* promoter methylation and GPNMB expression, IHC analysis of GPNMB protein expression and the corresponding results of methylation levels by pyrosequencing on the same tissues from colorectal cancer (*n* = 20), advanced adenoma (*n* = 48), and normal (*n* = 20) cases were combined. We showed high prevalence of *GPNMB* promoter methylation in advanced stages CRC (stage III-IV). The absence or weak staining at these stages (Table 4) suggest that there might be a correlation (43–78%) between the methylation and expression variables. Thus, a nonparametric measure of statistical dependence-Spearman’s rho (ρ)- between the two variables (level of methylation and intensity of expression) in normal, advanced adenoma, and CRC cases were computed. We used standard 2X2 table for presenting distribution of two categorical variables since it presents the correlation between two variables (methylation level as continues and expression as ordinal) for three groups. The correlation coefficient for all three types of group cases was negative (Table 5). However, in the normal group this correlation was not significant (*P* = 0.6). In advanced adenoma (*P* = 0.05) and CRC (*P* = 0.035), the correlation was statistically significant (Table 5). The negative correlation indicates that an increase of methylation level in samples leads to a decrease in expression. Therefore, an inverse relationship between the increase in methylation and decrease in expression in advanced adenoma (ρ = − 0.28) and CRC was established (ρ = − 0.47) (Table 5).

**Table 5:** Correlation table (Spearman correlation coefficient). There was significant negative correlation between methylaation level and expression in advanced adenoma and CRC groups

Groups	Samples	ρ	*P* value
Normal	20	−0.12	*P* = 0.6
Advanced adenoma	48	−0.28	*P* = 0.05
Colon cancer	20	−0.47	*P* = 0.035

No significant difference between methylation level and expression was observed in normal group

### Effect of GPNMB overexpression in colonic cell lines

Since *GPNMB* gene seems to play a role in the development and progression of CRC, and its role has not been well defined, a functional analysis was performed. An expression vector harboring the full length *GPNMB* cDNA (pEF1-GPNMB) was stably transfected into HCT116 cell line. As negative control, the empty vector (pEF1) was transfected into the same cell line. HCT116 cells transfected with pEF1 (mock) or pEF1-GPNMB, were grown for 2 weeks in media containing G418 for transfects’ selection. In the culture dishes, HCT116 cells with the empty vector grew faster when compared to the GPNMB expressing HCT116 transfected cells.

### GPNMB expression reduced cell proliferation in HCT116 cell line

**Fig. 5:**
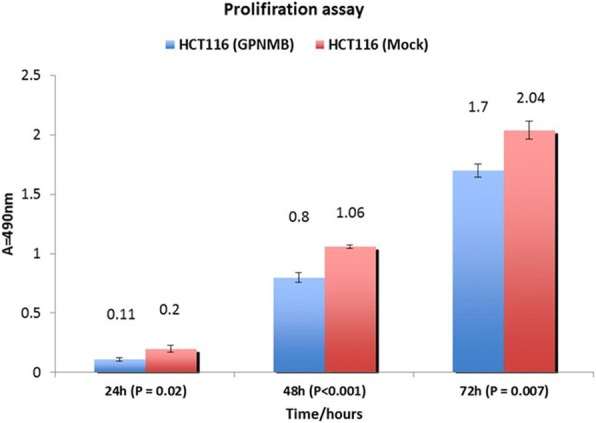
Proliferation data is expressed as mean number of GPNMB-transfected cells relative to control cells transfected with empty vector (pEF1). Three independent experiments were performed (*P* = 0.02, *P* < 0.001, and *P* = 0.007; two-sided Student t test). In comparison with mock control transfects, transfection with GPNMB cDNA significantly decreased cell proliferation

For proliferation studies of HCT116 cells transfected with empty vector (pEF1) and the GPNMB cDNA vector, cells were seeded onto 96-well plates (5000 cells per well), and cell numbers were counted 24, 48, and 72 h later (six wells per time point). At different incubation periods, cell proliferation was reduced in a statistically significant manner when GPNMB was expressed [(24 h, *P* = 0.02), (48 h, *P* < 0.001), and (72 h, *P* = 0.007)] (Fig. 5).

### GPNMB reduced cell migration and invasion in HCT116

**Fig. 6:**
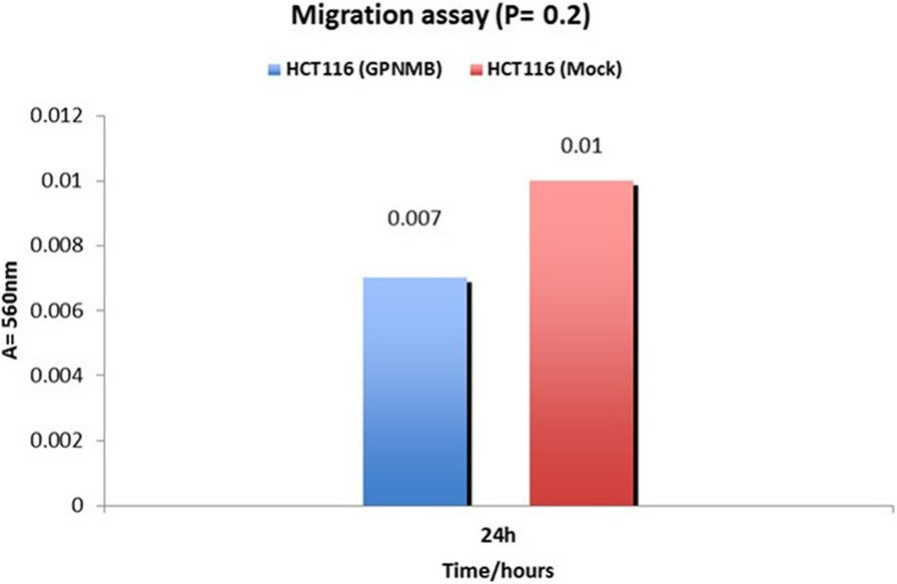
Effect of GPNMB on cell migration. Transfection of GPNMB had reduction effect on HCT116 cell migration compared with control-transfected cell in two independent experiments as determined on transwell membranes not coated with matrigel (8 μm pore size). The bar chart shows the mean number intensity of cell migration of each experiment. (*P* = 0.2; two-sided Student’s t test). When compared with control transfects, transfection with GPNMB decrease cell migration but was not significant

**Fig. 7:**
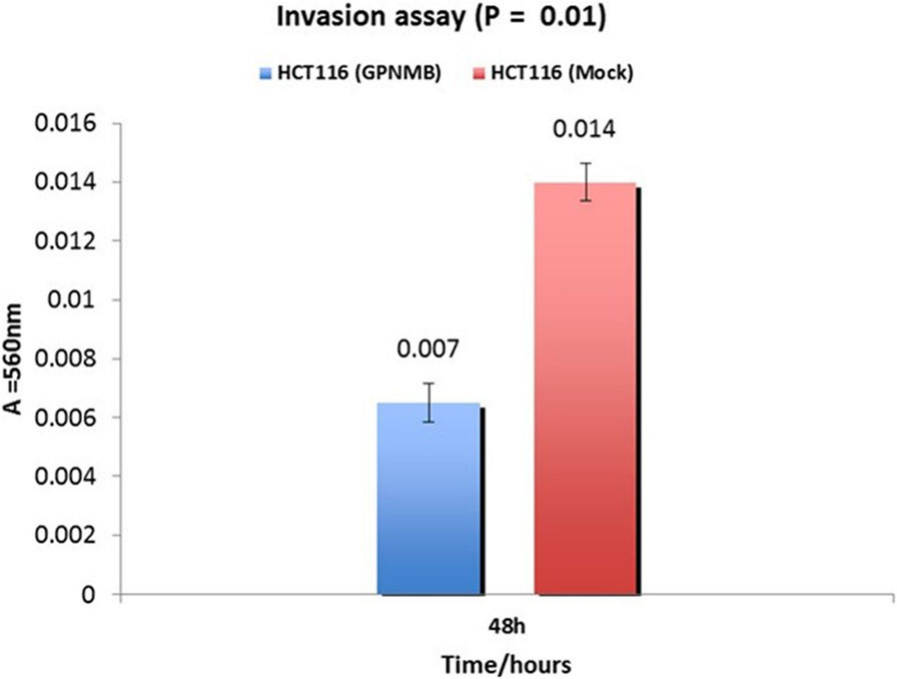
GPNMB transfection’s effect on invasion. Invasion showed 2.2-fold decrease through matrigel-coated transwell polycarbonate membranes (8 μm pore size) compared with control transfects. The bar chart shows the mean number of cell invasiveness of four independent experiments (*P* = 0.01, two side Student t test). Mock: negative control, empty vector (pEF1); and GPNMB expressing HCT116 cell line

Migration and invasion assays were performed using HCT116 cell line. Cell migration was reduced 1.43 fold in GPNMB overexpressing cells compared to the mock-transfected cells (Fig. 6). However, this change was not statistically significant (*P* = 0.2). GPNMB transfects displayed reduced invasion through matrigel-coated transwell membranes compared with control transfects (Fig. 7). Compared with control cell transfected with empty vector, transfection with GPNMB significantly reduced invasion through matrigel-coated transwell polycarbonate membranes (*P* = 0.01). Taken together, these data suggest that GPNMB exhibits tumor suppressive effects in human colorectal cancer cells.

## Discussion

It is now well recognized that along with genetic mutations, epigenetic mechanisms are also involved in cancer development [16, 22, 23]. The methylation of CpG islands, located in the promoter of tumor suppressor genes can lead to a complete or partial down regulation of expression. This model of silencing of tumor suppressor genes, has been defined in numerous types of cancer genes, nevertheless gene methylation does not always result in gene expression suppression as this is the result of the sum of many regulatory processes at once [22, 24].

Our present methylation results showed a statistically significant difference in the frequency of *GPNMB* promoter methylation between colorectal cancer and normal colon mucosa. Methylated *GPNMB* was found in 95% (19/20) CRC and 20% (4/20) of normal samples. *GPNMB* promoter methylation has been shown in both distal (often associated with chromosomal instability [25]) and proximal CRC (often associated with microsatellite instability) [26]. Since our goal is to identify early markers of neoplastic transformation in AAs, we analyzed the methylation status of *GPNMB* in pre-cancerous samples. The investigation of *GPNMB* promoter methylation in adenoma and advanced adenoma revealed 76% (16/21) and 94% methylation frequency (45/48), respectively. Interestingly, the difference of methylation frequency between adenoma and advanced adenoma was statistically significant. In addition, we found a statistically significant methylation frequency in adenoma and advanced adenoma when compared with normal mucosa. This result showed a substantial difference of average methylation levels in CpG sites of *GPNMB* with stepwise increase of methylation from normal colon mucosa to early and advanced adenomas (Table 2). This outcome suggests that *GPNMB* methylation takes place early in the adenoma-carcinoma sequence. Secondly, in the sequence from adenomas to carcinoma, GPNMB pyrosequencing methylation levels increased from 31.9% in non-advanced (< 1 cm tubular adenoma) to 42.6% in advanced adenoma (> 1 cm tubulovillous or villous adenoma) that might lead to faster progression to high potential malignant lesions. Indeed, our analysis of 3 advanced adenomas and their matched normal DNA revealed higher methylation levels in the lesions pointing to a role of GPNMB methylation in the neoplastic process. In addition, the non-significant difference of DNA methylation between advanced adenomas and CRC samples in this study is of great clinical and pathological implications. Indeed, this finding might be exploited to define adenomas with high carcinogenic potential. DNA methylation is not taking place only at early stages of tumorigenesis, but is likewise occurring in cancerous lesions as well [22]. The level of GPNMB methylation was variable at different stages of colon cancer. It was lower at early CRC stages and higher at advanced ones. It is noteworthy that this marker was first defined as silenced in metastatic melanoma cell lines when compared to non-metastatic ones. As such, the profile of this gene in melanoma seems to apply to CRC as well.

Our results from the IHC analysis of TMA indicated a reduction of GPNMB protein levels in adenoma and tumor tissues (both epithelial and stroma). It is tempting to speculate that the increase in *GPNMB* methylation in adenoma and tumor cases is directly responsible for the reduced GPNMB expression. However, hypermethylation of a specific gene is not always the sole process implicated in a given gene’s expression. This expression is the result of the cumulative effects of several DNA expression processes that are occurring simultaneously. The expression levels of GPNMB in human colon cancer cell lines (SW480, RKO, HCT116, and Colo320) were shown to decrease with higher levels of methylation [27] pointing to a potentially dominant methylation effect on GPNMB expression.

In order to further characterize factors affecting GPNMB expression in normal, advanced adenoma, and cancer samples, the IHC staining intensity for GPNMB was computed, and the difference between these three groups was found to be not significant even though there was a relatively stronger staining for normal specimens compared to moderate and weak staining in adenoma and cancer cases, respectively. Expression levels of GPNMB at different cancer stages were analyzed and substantially lower level of expression in advanced stage of colon cancer cases was observed. The data we presented here clearly display that GPNMB protein level significantly correlated with advanced cancer stages. Such relationship between higher stages and lower levels of GPNMB protein may be consistent in part with the fact that GPNMB protein could be involved in growth delay and reduction of CRC progression into advanced stages. Thus, lower expression levels of GPNMB might account for higher aggressiveness and fast progression of colon tumors. In other words, the loss of GPNMB that encodes a type I transmembrane glycoprotein located on the cell surface and involved in cell-to-cell adhesion likely leads to tissue disruption and cells acquiring invasive and migratory potential. It has been reported that hypermethylation in the promoter results in expression silencing of a number of genes such as tumor suppressor genes involved in apoptosis and DNA repair [22]. However, we found that in all colon samples included (cancer, advanced adenoma, and normal mucosa), no significant association between mean level of methylation in all 13 CpG sites on *GPNMB* promoter and expression was observed. Other mechanisms besides methylation might be involved. Indeed, Ripoll et al. reported that *GPNMB* is regulated by Microphthalmia transcription factor (MITF) in osteoclasts [28]. In addition, Mann et al. found that *GPNMB* expression is reduced by miR-155 in osteoclast, although the miR-155 binding sites apparently do not seem to exist in *GPNMB* 3’UTR [29].

Various studies indicating that promoter methylation of particular genes, regardless of their expression, may have a promoting role in colon oncogenic transformation, emphasizing the utility of DNA methylation as a marker to identify patients at risk for cancer recurrence [30]. As an example, the level of APC protein in colon cancer metastatic implants in liver shows no difference from normal colon. Yet, the methylation level in liver metastases is higher than local CRC, which can be helpful to predict and identify the hepatic metastasis of CRC [31]. It is worth noting that a correlation between higher methylation of *GPNMB* promoter at more advanced cancer stages samples was observed. This could be capitalized on by using high incidence methylated *GPNMB* in advanced adenoma as a surrogate marker to identify to patients with high risk for malignancy.

Research studies of GPNMB expression in specific cancer cells require careful evaluation because the outcomes seem to be context specific and dependent on tissue types [32]. GPNMB was originally evaluated in melanoma cell lines with low metastatic potential [19]. GPNMB expression has also been described in bladder, heart, lung, and small intestine [32]. The molecular characterization of GPNMB and the normal role of this protein in osteoblasts’ maturation and differentiation have been investigated [33]. Tsui et al. reported that re-expression of GPNMB in prostate cancer cell lines leads to a decrease in invasion and proliferation in-vitro and tumor development in-vivo. They suggested that *GPNMB* plays a key role as tumor suppressor in prostate carcinoma cells [32]. However, different studies have indicated that GPNMB is highly expressed in dendritic cells, hepatocellular carcinoma, gliomas, squamous cell lung carcinoma, melanoma, soft tissue tumors, and cancer of the breast, stomach, and pancreas [34–38]. In human breast cancer, there is still a debate between different studies whether *GPNMB* acts as a tumor suppressor or an oncogene [39, 40]. These studies pointed out the potential complex role of GPNMB in tumorigenesis, making it mandatory to establish precise spatial (different tissue and organ types) and temporal (sequence from normal to cancer and within different cancer stages) studies for an accurate functional analysis. An expanding number of studies are evaluating the mechanism of GPNMB in cancer biology, determining its role and exploring the relationship of its expression and pro-invasive pro-metastatic phenotype in different malignancies [41].

Regarding the role of GPNMB in the development and progression of CRC, functional analysis of this gene was performed. *GPNMB* re-expression, in HCT116 cell line was induced through a GPNMB-cDNA vector transfection. Re-expression of *GPNMB* in HCT116 cells showed a significant reduction in cell proliferation, and invasion compared to mock control transfects. The *GPNMB* re-expression in HCT116 cells resulted in a decrease in migration ability in comparison with mock-transfected controls. Overall, these studies are indicative of tumor suppressor activities of *GPNMB* in CRC cell lines experiments.

Prostate cancer experimental data also demonstrated a tumor suppressor activity for *GPNMB* [32]. On another hand, Rose et al. identified a high level of GPNMB protein in metastatic breast cancer, such as bone metastasis [39]. We reported that reduced *GPNMB* gene expression was correlated with high-grade tumor and metastasis in CRC.

The expression of GPNMB in prostate cancer cell lines and animal models results in the activation of N-myc downstream-regulated gene 1 (Ndrg1) in-vitro and in-vivo [32]. Interestingly, the NDRG1 contributes in TGF- β1 and Wnt-β-Catenin signaling pathways that usually silenced in metastatic colon cancers [42–44]. Overall, these studies indicate that upregulation of NDRG1 by GPNMB can inhibit TGF-β-induced EMT and eventually preventing metastasis.

Tsui et al. reported that over expression of p53 leads to GPNMB expression in prostate cancer cell line [32], which is in agreement with data of previous studies indicating the contribution of GPNMB in cell cycle regulation via the p53 pathway [45]. The *p53* gene is involved in regulation of the cell cycle, apoptosis, and the initiation of cell aging process [46, 47], thus prevents abnormal overgrowth of colonic mucosa. Zhang et al. also reported that inhibition of NDRG1 up-regulation by p53 induction prevents intestinal epithelial cell (IEC) proliferation [48]. In combination with our proliferation assay results, it was shown that over-expression of GPNMB in HCT116 cell line diminished proliferation in colon cancer cells, however corresponding signaling pathway in colon cancer need to be explored in GPNMB expressing and non-expressing colon cancer cell lines.

## Conclusion

We have established in this study the tumor suppressor status of GPNMB gene in colon oncogenic transformation. This new marker not only has the power to show potentially carcinogenic adenomas but also cancer lesions with metastatic potential. Its methylation status can be used as a biomarker to assess the progression of colorectal lesions.

## Abbreviation

AA: African Americans
AD: Advanced Adenoma
CRC: Colorectal Cancer
GPNMB: GlycoProtein Non-metastatic Melanoma B
IHC: Immunohistochemistry
TMA: Tissue Microarrays
